# Chloramphenicol and tetracycline decrease motility and increase invasion and attachment gene expression in specific isolates of multidrug-resistant *Salmonella enterica* serovar Typhimurium

**DOI:** 10.3389/fmicb.2014.00801

**Published:** 2015-01-30

**Authors:** Brian W. Brunelle, Bradley L. Bearson, Shawn M. D. Bearson

**Affiliations:** ^1^Food Safety and Enteric Pathogens Research Unit, National Animal Disease Center, Agricultural Research Service, United States Department of AgricultureAmes, IA, USA; ^2^Agroecosystems Management Research Unit, National Laboratory for Agriculture and the Environment, Agricultural Research Service, United States Department of AgricultureAmes, IA, USA

**Keywords:** antibiotics, attachment, chloramphenicol, fimbriae, invasion, motility, *Salmonella*, tetracycline

## Abstract

*Salmonella enterica* serovar Typhimurium is one of the most common serovars isolated from humans and livestock, and over 35% of these isolates are resistant to three or more antibiotics. Multidrug-resistant (MDR) *Salmonella* is a public health concern as it is associated with increased morbidity in patients compared to antibiotic sensitive strains, though it is unknown how the antibiotic resistant isolates lead to a more severe infection. Cellular invasion is temporally regulated in *Salmonella* and normally occurs during late-log and stationary growth. However, our previous work determined that a 30 min exposure to a sub-inhibitory concentration of tetracycline can induce the full invasion phenotype during early-log growth in certain MDR *S*. Typhimurium isolates. The current study examined whether sub-inhibitory concentrations of other antibiotics could also induce the invasiveness in the same set of isolates. Ampicillin and streptomycin had no effect on invasion, but certain concentrations of chloramphenicol were found to induce invasion in a subset of isolates. Two of the isolates induced by chloramphenicol were also inducible by tetracycline. RNA-seq analyses demonstrated that chloramphenicol and tetracycline both down-regulated motility gene expression, while up-regulating genes associated with attachment, invasion, and intracellular survival. Eleven fimbrial operons were up-regulated, which is notable as only three fimbrial operons were thought to be inducible in culture; six of these up-regulated operons have been reported to play a role in *Salmonella* persistence in mice. Overall, these data show that the normal progression of the genetic pathways that regulate invasion can be expedited to occur within 30 min due to antibiotic exposure. This altered invasion process due to antibiotics may play a role in the increased intensity and duration of infection observed in patients with MDR *Salmonella*.

## Introduction

*Salmonella* is a highly prevalent bacterial food-borne disease. Annually in the United States, human salmonellosis is estimated to cause over 1 million cases, 19,000 hospitalizations, 350 deaths, and $2.6 billion in associated costs (Economic Research Service, 2009; Scallan et al., 2011). *Salmonella enterica* serovar Typhimurium is one of the most commonly isolated salmonellae in both humans and livestock, and there is a high incidence of antibiotic resistance among these isolates. According to a 10-year average from the National Antimicrobial Resistance Monitoring System (NARMS), isolates resistant to three or more antibiotics are frequent among *S*. Typhimurium from humans (35%), chickens (41%), cattle (63%), and swine (78%) (CDC, 2012). The NARMS report also lists that the four most common antibiotics to which human isolates of *S*. Typhimurium are resistant are tetracycline (33% of isolates), ampicillin (31%), streptomycin (31%), and chloramphenicol (24%). Resistance to these antibiotics is mediated by antibiotic-specific mechanisms that can be acquired via horizontal transfer (*tetA*, *floR*, *strA*, etc.). Multidrug-resistant (MDR) *Salmonella* has emerged as an important food safety concern and is associated with increased morbidity in humans compared to antibiotic sensitive strains (Molbak, 2005; Varma et al., 2005). However, little is known why MDR *Salmonella* is more virulent as it is not due to an intrinsically higher capacity to invade eukaryotic cells *in vitro*; in fact, MDR *Salmonella* has been reported to be less invasive in tissue culture experiments compared to antibiotic-sensitive strains (Carlson et al., 2000). The increased virulence capacity may be triggered by effectors in the environment, the host, or both.

A temporal progression of regulated expression occurs in *Salmonella* for genes associated with virulence and cellular invasion (Ibarra et al., 2010; Saini et al., 2010; Fabrega and Vila, 2013). During early-log growth, *Salmonella* express flagella and are motile, but have limited-to-no invasive capabilities. As late-log and stationary phases are reached, motility is down-regulated, and genes associated with attachment, invasion, and intracellular survival are up-regulated. Attachment is mediated by fimbriae, and *S*. Typhimurium encodes up to 13 fimbrial operons (McClelland et al., 2001). Invasion genes are located in *Salmonella* Pathogenicity Island 1 (SPI-1), which encodes a type three secretion system (TTSS) and effector molecules that promote cellular entry (Lostroh and Lee, 2001). SPI-2 also encodes a TTSS and effector molecules, but these and the genes expressed in SPI-3 promote *Salmonella* intracellular survival (Kuhle and Hensel, 2004).

Antibiotics can affect the regulation of cellular processes in bacteria (Goh et al., 2002; Kuroda et al., 2007; Shen et al., 2008; Deneve et al., 2009; Morita et al., 2014). For example, florfenicol can induce adherence of *Staphylococcus aureus* (Blickwede et al., 2005), and tetracycline can increase TTSS expression and cytotoxicity in *Pseudomonas aeruginosa* (Linares et al., 2006). However, the majority of bacteria in such studies are resistant to antibiotics due to intrinsic factors (such as mutations) or efflux pumps that work against a suite of antimicrobials. Antibiotic-specific genes often provide a much higher level of resistance compared to the non-specific antimicrobial pumps and can allow growth at an antibiotic concentration that might otherwise be inhibitory to sensitive bacteria. We previously characterized the effect different sub-inhibitory concentrations of the antibiotic tetracycline had on the invasiveness of eight MDR *S*. Typhimurium isolates that encoded tetracycline-specific resistance efflux pumps (Brunelle et al., 2013). We found that a subset of the isolates had an induced invasion phenotype during early-log growth phase in response to 16 μg/ml of tetracycline. The induced invasiveness was not observed with lower concentrations of tetracycline during early-log growth (1 and 4 μg/ml), nor did tetracycline enhance invasion in any isolate during late-log growth at any antibiotic concentration tested. The goals of the current study were to establish if ampicillin, chloramphenicol, or streptomycin can also induce invasion in the same isolates, and to assess transcriptional changes for genes associated with the virulence functional groups (motility, SPI-1, SPI-2, SPI-3, and attachment) due to antibiotic exposure.

## Materials and methods

### Isolates

Eight isolates of *Salmonella enterica* serovar Typhimurium phage types DT104 (530, 290, 360) and DT193 (1434, 5317, 752, 1306, 4584) originally from cattle, and previously tested for differences in invasiveness after exposure to tetracycline (Brunelle et al., 2013), were selected for study. These isolates were cultured on solid media with antibiotic concentrations based on CLSI breakpoints, and it was identified that they are resistant to ampicillin (32 μg/ml), chloramphenicol (32 μg/ml), streptomycin (64 μg/ml), and tetracycline (16 μg/ml), but are sensitive to gentamicin (16 μg/ml).

### Characterization of antibiotic resistance genes

Primers (Table 1) were used to amplify and detect the most common resistance genes in *Salmonella* for the following antibiotics: ampicillin (*bla_CMY_*, *bla_OXA_*, *bla_PSE_*, *bla_TEM_*, and *bla_SHV_*), chloramphenicol (*cat*, *cml*, and *floR*), and streptomycin (*aadA2*, *strA*, and *strB*). Tetracycline resistance genes in these isolates (*tetA*, *B*, *C*, *D*, and *G*) were characterized previously (Brunelle et al., 2013). DNA was obtained by boiling a single colony from each isolate in 30 μl water. Each 25 μL PCR reaction contained 1.5 μl DNA, 1.5 units of Taq polymerase (Promega), 1x PCR buffer with 1.5 mM MgCl_2_, 1 mM each dNTP, and 0.8 μM of each primer. Amplification conditions were: 94°C for 1 min; 35 cycles of 94°C for 30 s, 56°C for 30 s, 72°C for 30 s; 72°C for 2 min; 4°C hold. Amplifications were done in duplicate and products were visualized on 2% NuSieve agarose gels (Cambrex, Rockland, ME).

**Table 1:** **Primers used for identification and real-time PCR**.

**Gene**	**Forward primer (5′-3′)**	**Reverse primer (5′-3′)**	**References**
16S rRNA	CGGGGAGGAAGGTGTTGTG	GAGCCCGGGGATTTCACATC	Chang et al., 1997
*hilA*	CGCTGGCAGAATGCTACCTC	AGCCCCAGTAATCCTAAAGCTTG	Brunelle et al., 2011
*prgH*	GCTCTTTCTTGCTCATCGT	ATCTCTATCTGGCTGGATACCT	Brunelle et al., 2013
*invF*	ATGTGAAGGCGATGAGTAAC	GCTGCTGAATAGTGTAGAAGG	Brunelle et al., 2013
*floR*	TTCGGTCAAGGTTCTGGACCAGTT	TGGACATAAGCCTGTTCGGTTGGT	Golding et al., 2007
*cat*	GCAAGATGTGGCGTGTTAC	GGGGGCGAAGAAGTTGTC	This study
*cml*	CGGCTGAAGTCTTTCTGG	CGACCTGCGTAAATGTCAC	This study
*aadA2*	ATGACCTTATGGAGGCTTCGGCTT	TGCCATTCTCCAAATTGCAGCTCG	Golding et al., 2007
*strA*	GGTAAGAAGTCGGGATTGAC	CACAGCCTATCGGTTGATC	This study
*strB*	CGCCATACCAGATAGTCG	CTTTTCCAGCCTCGTTTG	This study
*bla_CMY_*	ATGATGAAAAAATCGTTATGC	TTGTAGCTTTTCAAGAATGCGC	Eckert et al., 2004
*bla_OXA_*	ATGAAAAACACAATACATATC	AATTTAGTGTGTTTAGAATGG	Weill et al., 2006
*bla_PSE_*	CGGCGGGATGGAACATT	GCTGTAATACTCCGAGCACCAA	Golding et al., 2007
*bla_TEM_*	ATGAGTATTCAACATTTCCG	CCAATGCTTAATCAGTGAGG	Eckert et al., 2004
*bla_SHV_*	TTATCTCCCTGTTAGCCACC	GATTTGCTGATTTCGCTCGG	Weill et al., 2006
*tetA*	GCTACATCCTGCTTGCCTTC	CATAGATCGCCGTGAAGAGG	Weir et al., 2008
*tetC*	GCATAAACCAGCCATTGAG	GGTAAACGCCATTGTCAG	Brunelle et al., 2013
*tetD*	GATGTGGCGAATAAAGCG	CCAGTGTGACCCCTGTTAC	Brunelle et al., 2013
*tetG*	CCTTGCAGGCAATGCTCTCAAACA	AGATTGGTGAGGCTCGTTAGCGTT	Golding et al., 2007

### Characterization of antibiotic concentrations that inhibit growth

The antibiotic concentration that inhibited growth in broth culture for each isolate was determined. Overnight cultures were diluted 1:200 in LB and grown to early-log phase (OD_600_ = 0.15) at 37°C with agitation before adding serial 2-fold dilutions of ampicillin, chloramphenicol, or streptomycin (0, 2-512 μg/ml). All conditions were done in triplicate. Cultures were continuously shaken at 37°C, and growth measurements (OD_600_) were taken every hour for 24 h using a Bioscreen C instrument (Growth Curves LTD, Raisio, Finland). Growth curves on serial dilutions of tetracycline were established previously (Brunelle et al., 2013).

### Culture conditions

For each experiment, *Salmonella* was plated on Lennox L (LB) agar plates (Invitrogen, Carlsbad, CA), and a single colony was selected and grown in LB broth with agitation for 6 h at 37°C. The 6 h culture was diluted 1:1000 in fresh LB broth and grown with agitation at 37°C overnight. The overnight culture was diluted 1:200 in fresh LB broth and divided into 16 × 100 mm glass tubes. Cultures were grown to early-log (OD_600_ = 0.15) with agitation at 37°C before antibiotic addition. An aliquot was taken for RNA analysis from each culture and placed in RNAProtect (QIAGEN, Germantown, MD). Ampicillin, chloramphenicol, and streptomycin were added to separate tubes for a final concentration of 0 (control), 16, 32, 64, and 128 μg/ml, and these were incubated with agitation at 37°C for 30 min; tetracycline was assayed at only 0 and 16 μg/ml. Aliquots for RNA analysis were taken from each bacterial culture and placed in RNAProtect. An additional aliquot was taken from each culture for a cell culture invasion assay. All experiments were performed four separate times.

### *Salmonella* invasion assays

The aliquots of *Salmonella* cultures taken following the 30 min incubation with and without antibiotics were centrifuged at 16,000 × g for 2 min, and the pellets were re-suspended in fresh LB broth to remove the antibiotic. Invasion assays were performed with technical replicates for each biological replicate using a gentamicin protection assay in HEp-2 cells at a multiplicity of infection of ~40 as previously described (Elsinghorst, 1994). Percent invasion was calculated by dividing the CFU/ml recovered by the CFU/ml originally added to the cells. The significance of the differences in invasion was determined by a one-way repeated measures ANOVA with Dunnett's post-test to assess pair-wise differences between the no-antibiotic control and the other sample conditions using GraphPad Prism 5 (San Diego, CA). Differences were considered significant if *P* < 0.05. Each isolate has an intrinsically different invasion rate when grown without antibiotics; therefore the invasion data for each isolate was normalized to its respective control for graphical representation.

### Real-time PCR assays

RNA was isolated using the RNeasy Mini Kit (QIAGEN, Germantown, MD), and genomic DNA was removed by the Turbo DNase DNA-free kit (Ambion, Austin, TX) according to the product directions. Total RNA was quantitated on a Nanodrop ND-1000 spectrophotometer (Thermo Scientific, Wilmington, DE). Reverse transcription was carried out by the Applied Biosystems High capacity cDNA reverse transcription kit on total RNA with random primers (Life Technologies, Grand Island, NY), and two technical replicates were performed for each of the three biological replicates. Real-Time PCR was performed in a Bio-Rad CFX96 Real-Time PCR Detection System (BioRad Laboratories, Hercules, CA) using the SYBR Green Master Mix (Applied Biosystems, Foster City, CA). Primer sets were used to evaluate the 16S *rRNA*, *hilA*, *floR*, *tetA*, *tetC*, *tetD*, and *tetG* transcripts (Table 1). For control assays, reverse transcriptase was not added to parallel mixtures for each sample. The amplification cycle conditions were as follows: 95°C for 10 min; 40 cycles of 95°C for 15 s, 55°C for 30 s, 72°C for 30 s; melting curve analysis from 65°C to 95°C. Raw fluorescence data was analyzed with the LinRegPCR software, and amplification efficiencies and cycle threshold (C_T_) values were determined by linear regression in a Window of Linearity for each primer set (Ramakers et al., 2003). Expression differences were calculated using the C_T_ value and efficiency for each locus by the Pfaffl method (Pfaffl, 2001), where the 16S gene was the reference gene and the pre-antibiotic sample was the control condition for each isolate at each antibiotic concentration. Values were log_2_ transformed, and GraphPad Prism 5 was used to perform a one-way repeated measures ANOVA with Dunnett's post-test to assess pair-wise differences between the no-antibiotic control and the other sample conditions. Differences were considered significant if *P* < 0.05.

### RNA transcriptome sequencing

Isolates 1434, 5317, 752, 4584, and 530 were selected for RNA-seq analysis to identify changes in virulence gene expression due to tetracycline (0 and 16 μg/ml) and chloramphenicol (0, 32, and 64 μg/ml) exposure. RNA from three biological replicates of each isolate and condition were used for sequencing. Quality and integrity of the RNA was assessed with the 2100 Bioanalyzer (Agilent Technologies, Santa Clara, CA). The RiboZero kit was used to deplete the ribosomal RNA (rRNA) sequences according to the manufacturer's instructions (Epicentre, Madison, WI). The 2100 Bioanalyzer was used to verify removal of the rRNA. Libraries were constructed using TruSeq RNA Sample prep kit and were sequenced on a HiSeq 2500 employing a 100-cycle run (Illumina Inc., San Diego, CA) at the Iowa State University DNA core facility.

### RNA-seq data analyses

CLC Genomics Workbench v7.0 was used to import the Illumina sequence data. Failed reads were removed prior to analysis. The *S*. Typhimurium SL1344 genome [GenBank: FQ312003] and three plasmids [GenBank: HE654724-26], as well as a MDR plasmid from *S*. Heidelberg [GenBank JN983048], were used as references for mapping with the following parameters: 2 maximum mismatches, 90% minimum length fraction, 80% minimum similarity fraction, and 10 maximum hits per read. Differences in gene expression were calculated using EdgeR (total count filter cut-off = 5.0) with False Discovery Rate (FDR) corrected *P*-values (Robinson et al., 2010). Differences were considered significant if *P* < 0.05. Fold-changes for RNA-seq data described in the study are the weighted differences between groups based on counts per million calculated in EdgeR. Functional categories for genes associated with motility, attachment, SPI-1, SPI-2, and SPI-3 were manually assigned.

### Electron microscopy

*Salmonella* isolate 1434 was grown in LB to early-log growth phase followed by a 30 min antibiotic exposure to 64 μg/ml chloramphenicol, 16 μg/ml tetracycline, or no antibiotic (control) as described above. One ml of each culture was centrifuged at 1000 × g for 5 min, the supernatant was removed, and the pellet was re-suspended in 0.5 ml of 0.5% methyl cellulose solution in water (Mitani and Iino, 1968). Samples were prepared for negative stain examination by electron microscopy following previously described procedures (Doane and Anderson, 1987). Each sample was negatively stained with 2% phosphotungstic acid (pH 7.0) and examined with a FEI Tecnai G^2^ BioTWIN electron microscope (FEI Co., Hillsboro, OR) at the National Animal Disease Center Core Facility.

## Results and discussion

Our previous work investigated eight MDR *S*. Typhimurium isolates that were resistant to ampicillin, chloramphenicol, streptomycin, and tetracycline and demonstrated that a sub-inhibitory concentration of tetracycline induced the full invasive phenotype in a subset of these isolates during early-log growth phase (Brunelle et al., 2013). In order to determine if other antibiotics could induce invasiveness in the same eight isolates, the current study used invasion assays to test their response to sub-inhibitory concentrations of ampicillin, chloramphenicol, and streptomycin. Real-time PCR and RNA-seq were used to assess expression changes of genes associated with antibiotic resistance, as well as with the temporal regulation of the invasion phenotype.

### Characterization of antibiotic resistance

PCR was used to identify the presence of commonly encoded MDR *Salmonella* genes that mediate resistance to ampicillin (*bla_CMY_*, *bla_OXA_*, *bla_PSE_*, *bla_TEM_*, and *bla_SHV_*), chloramphenicol (*cat*, *cml*, and *floR*), and streptomycin (*aadA2*, *strA*, and *strB*). With the exception of *bla_TEM_* and *bla_OXA_*, all genes were present in some combination in the eight isolates (Table 2). The tetracycline resistance genes in these isolates were established previously (Brunelle et al., 2013). As expected, *bla_PSE_*, *aadA2*, and *tetG* were identified in all three DT104 isolates, but not in any of the DT193 isolates, as these resistance genes are typically chromosomally encoded on the *Salmonella* Genomic Island 1 (SGI-1) in DT104 (Boyd et al., 2001). Of note is that 4584 was the only isolate to lack the *floR* gene, as well as the only one to encode the *cat* gene.

**Table 2:** **Resistance gene profiles for the eight *S*. Typhimurium isolates in the study**.

**Isolate**	**Tetracycline**						**Chloramphenicol**			
	***tetA***	***tetB***	***tetC***	***tetD***	***tetG***	**MIC μg/ml**	***floR***	***cat***	***cml***	**MIC μg/ml**
1434	+	−	−	−	−	256	+	−	−	128
5317	+	−	−	−	−	256	+	−	+	128
752	+	−	−	−	−	256	+	−	−	128
1306	+	+	+	+	−	256	+	−	+	128
4584	−	+	+	+	−	256	−	+	−	>512
530	−	−	−	−	+	64	+	−	−	128
290	−	−	−	−	+	64	+	−	−	128
360	−	−	−	−	+	64	+	−	−	256
**Isolate**	**Ampicillin**						**Streptomycin**			
	***CMY***	***PSE***	***SHV***	***OXA***	***TEM***	**MIC μg/ml**	***aadA2***	***strA***	***strB***	**MIC μg/ml**
1434	+	−	+	−	−	>512	−	+	+	>512
5317	+	−	+	−	−	>512	−	+	+	>512
752	+	−	+	−	−	>512	−	+	+	>512
1306	−	−	+	−	−	>512	−	+	+	>512
4584	−	−	+	−	−	>512	−	+	+	>512
530	−	+	+	−	−	>512	+	−	−	>512
290	−	+	+	−	−	>512	+	−	−	>512
360	−	+	+	−	−	>512	+	−	−	>512

**Figure 1:**
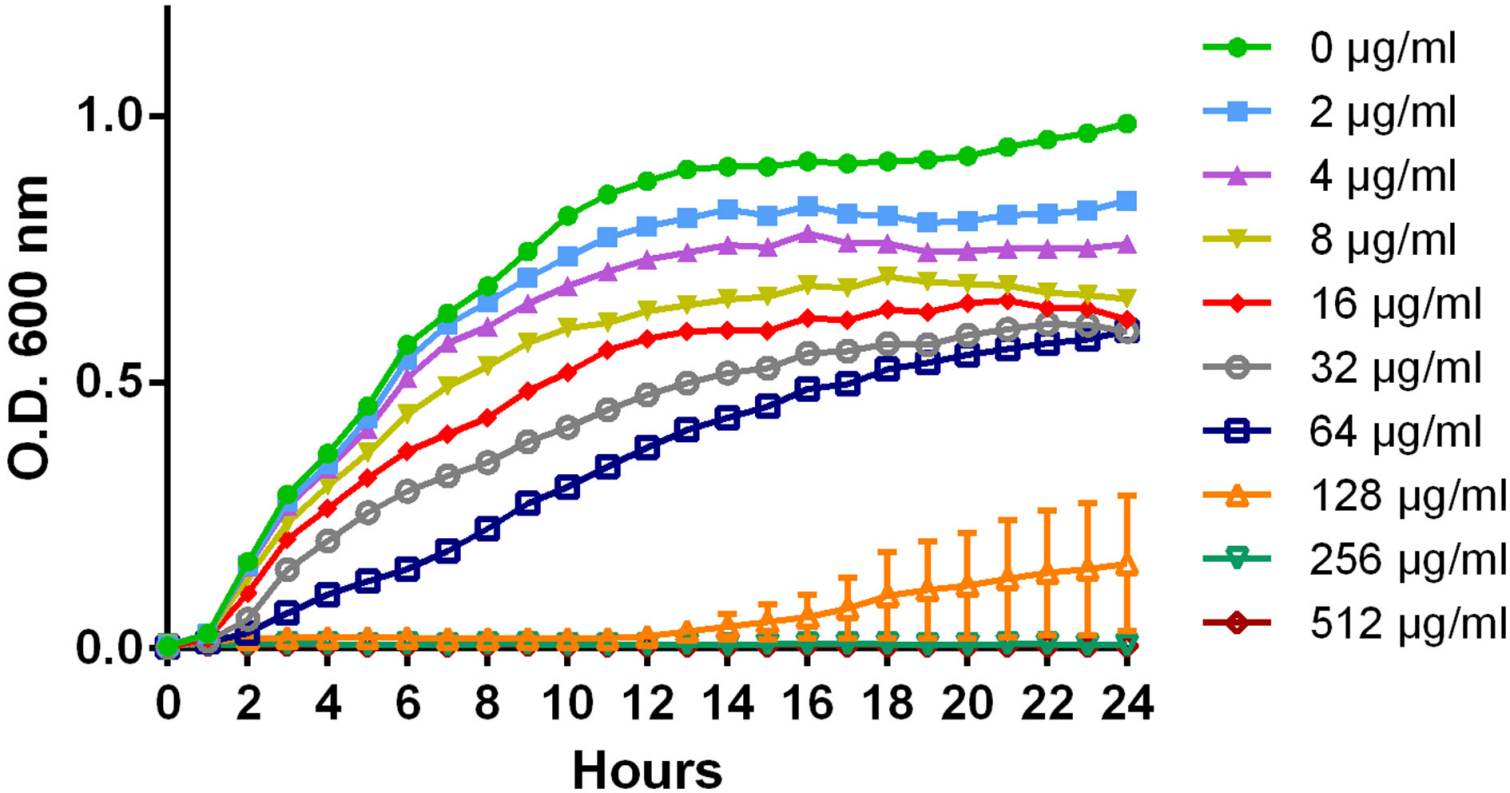
**Growth curve of multidrug-resistant *S*. Typhimurium isolate 1434 exposed to chloramphenicol**. Serial two-fold dilutions of chloramphenicol (0, 2-512 μg/ml) were added at OD_600_ = 0.15 to the eight isolates in the study to assess the effect chloramphenicol exposure had on growth. The growth curve of isolate 1434 is representative of the other isolates, with the exception of 4584.

The effect different concentrations (0, 2-512 μg/ml) of ampicillin, chloramphenicol, and streptomycin had on the growth of each isolate was determined (Table 2). For chloramphenicol, growth was inhibited at 128 μg/ml for six of the isolates, including isolate 1434 (Figure 1), and at 256 μg/ml for isolate 360 (Table 2). Isolate 4584 grew at 512 μg/ml, which is likely because it encodes the antibiotic-modifying enzyme chloramphenicol acetyltransferase (*cat*) instead of the efflux pumps *floR* or *cml* (Schwarz et al., 2004). The ampicillin and streptomycin resistance genes in the isolates encode modifying enzymes, and neither antibiotic inhibited growth at any of the concentrations that were tested.

### Chloramphenicol induces invasion in three isolates

**Figure 2:**
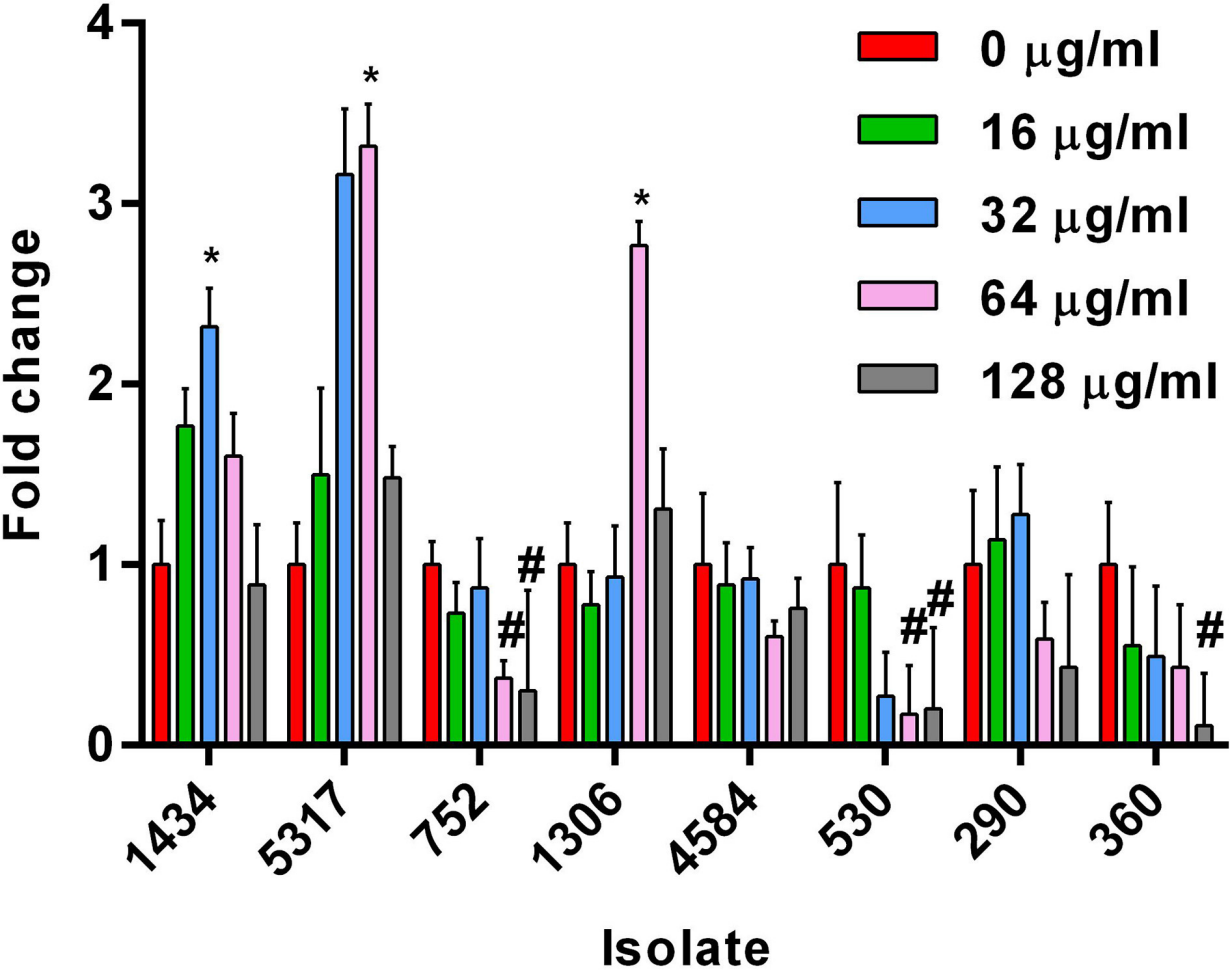
**Changes in cellular invasiveness after chloramphenicol exposure in *S*. Typhimurium isolates during early-log growth**. Invasion assays were performed using HEp-2 cells. *S*. Typhimurium isolates grown to early-log phase and exposed to chloramphenicol (0, 16, 32, 64, or 128 μg/ml) for 30 min. Changes in invasion were normalized to the control dose (0 μg/ml) for each isolate. The “^*^” indicates a significant increase based on the pre-normalized data, while “#” denotes a significant decrease (*P* < 0.05).

Based on the growth data, 0, 16, 32, 64, and 128 μg/ml of ampicillin, chloramphenicol, and streptomycin were chosen as the sub-inhibitory concentrations to test the effect each antibiotic had on invasion during early-log growth phase of the eight isolates. Chloramphenicol induced a significant increase in invasiveness at 32 or 64 μg/ml compared to the no-antibiotic control for three DT193 isolates (1434, 5317, and 1306; Figure 2). The chloramphenicol-induced invasion rates during early-log are similar to the maximum natural invasion rates for each isolate during late-log, which parallels the results observed for tetracycline (Brunelle et al., 2013) (data not shown). Isolates 1434 and 5317 were previously shown to have the invasion phenotype induced after exposure to 16 μg/ml tetracycline for 30 min during early-log growth. Several isolates had a significant decrease in cellular invasion at 64 μg/ml (752 and 530) and 128 μg/ml (752, 530, and 360) of chloramphenicol. Neither ampicillin nor streptomycin had an effect on the invasiveness of any of the isolates at any concentration tested (data not shown). Therefore, invasion was induced by two different antibiotics in a subset of isolates that utilize efflux pump-mediated resistance (*floR* and *tetA*), but not in the isolates that use enzyme-mediated resistance (*cat*, all ampicillin and streptomycin genes). However, several isolates that utilize TetA, TetG, or FloR efflux pumps did not have the induced invasion phenotype.

### Real-time PCR analyses

Real-time PCR was used to assess expression changes of *hilA*, *floR*, and the *tet* genes in all the isolates in response to the 30 min chloramphenicol exposure.

#### Differential expression of the hilA gene

**Figure 3:**
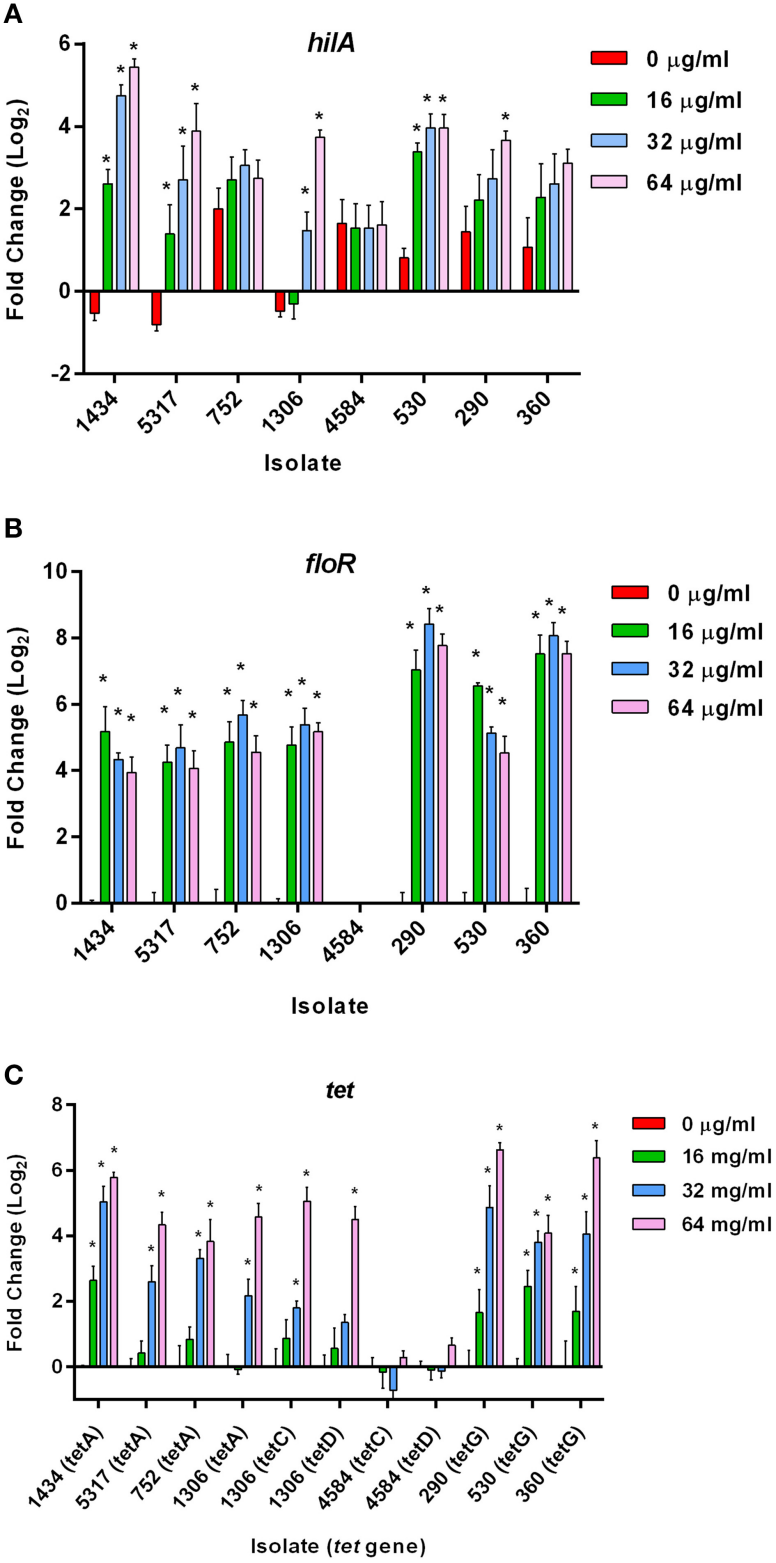
**Gene expression changes in *S*. Typhimurium at early-log growth after chloramphenicol exposure**. Real-time gene expression assays were performed on eight MDR *S*. Typhimurium isolates grown to early-log phase and exposed to different concentrations of chloramphenicol (0, 16, 32, and 64 μg/ml) for 30 min. The “^*^” denotes a significant change in expression over the 30 min compared to the control (pre-incubation) (*P* < 0.05). **(A)** Gene expression changes in the *hilA* gene. **(B)** Gene expression changes in the chloramphenicol-resistance *floR* gene (not present in isolate 4584). **(C)** Gene expression differences in the tetracycline-resistance genes in the eight isolates due to chloramphenicol exposure.

The HilA protein is the master regulator of SPI-1, and increases or decreases in expression of the *hilA* gene typically have a corresponding increase or decrease on invasion (Lee et al., 1992). However, increases in *hilA* expression due to chloramphenicol (Figure 3A) did not always correlate with increased invasiveness (Figure 2); this is highlighted in isolate 530 as 16, 32, and 64 μg/ml of chloramphenicol significantly increased *hilA* expression without any corresponding changes on invasion. This lack of congruence between increased *hilA* gene expression and increased invasiveness was previously observed when the isolates were exposed to tetracycline (Brunelle et al., 2013). No changes in *hilA* expression were detected due to either ampicillin or streptomycin exposure in any of the isolates, which is consistent with the invasion data (data not shown).

#### Differential expression of the floR gene

An increase in the expression of *floR* was significant at all concentrations of chloramphenicol in the seven isolates encoding the gene (Figure 3B). These data indicate that there is no direct association between the up-regulation of *floR* and the induced invasiveness in isolates 1434, 5317, and 1306. Therefore, *floR* may be necessary, but is not sufficient for the observed effect. This parallels previous findings that the increased expression of *tet* genes due to tetracycline exposure was not directly responsible for tetracycline-induced invasion (Brunelle et al., 2013).

#### Differential expression of the tetA,C,D,G genes

Expression changes of the tetracycline resistance genes *tetA*, *tetC*, *tetD*, and *tetG* were also analyzed in response to chloramphenicol (Figure 3C); *tetB* expression with or without antibiotic exposure was undetectable, and therefore was not included in the figure. Interestingly, *tetA*, *C*, *D*, and *G* were found to be up-regulated due to exposure to various concentrations of chloramphenicol (and in the absence of tetracycline) in most of the isolates. Only isolate 4584, which encodes *tetC*, *tetD*, and *cat* but lacks *tetA* and *floR*, did not have a change in expression of its *tet* genes. In contrast, the *tetC* and *tetD* genes in isolate 1306 were up-regulated at 64 μg/ml of chloramphenicol. Isolate 1306 encodes both *floR* and *tetA*, and it may be the presence of one or both of these two genes that is needed to co-regulate *tetC* and *tetD* expression after exposure to chloramphenicol. Additionally, *floR* is frequently adjacent to *tetA* in DT193 and *tetG* in DT104. Although these genes are in opposite orientations and would not be in the same operon, it could be the proximity of these genes that allows chloramphenicol to induce the *tet* genes independently of tetracycline. PCR confirmed the order and orientation of the *floR* and *tetA/G* genes in the isolates.

### RNA-seq identifies differential regulation of the virulence-associated functional groups due to antibiotic exposure

In order to assess if the early induction of the full invasive phenotype by chloramphenicol and tetracycline coincided with an acceleration of the temporally regulated virulence pathways, RNA-seq was used to evaluate differences in the expression of genes in the functional groups required for invasion. Five isolates were chosen to study: isolates 1434 and 5317 because they both have an increase in invasion after exposure to tetracycline and chloramphenicol, and both encode *floR* and *tetA*; isolates 752 and 530 because they do not have increased invasiveness due to antibiotic exposure, but encode *floR* and either *tetA* (752) or *tetG* (530); and isolate 4584 as it did not have a change in its invasion phenotype and lacks *floR*, *tetA*, and *tetG*. Genes associated with motility, invasion (SPI-1), intracellular survival (SPI-2 and SPI-3), and attachment were examined in isolates exposed separately to no antibiotic (control), 16 μg/ml of tetracycline, and 32 and 64 μg/ml of chloramphenicol for 30 min during early-log growth. The results for the samples exposed to 32 and 64 μg/ml of chloramphenicol were highly similar, so only the 64 μg/ml of chloramphenicol data are presented. Except for SPI-3, the data for isolate 4584 is not included below as there were no significant changes in any of the genes among the other functional groups.

#### Motility

**Figure 4:**
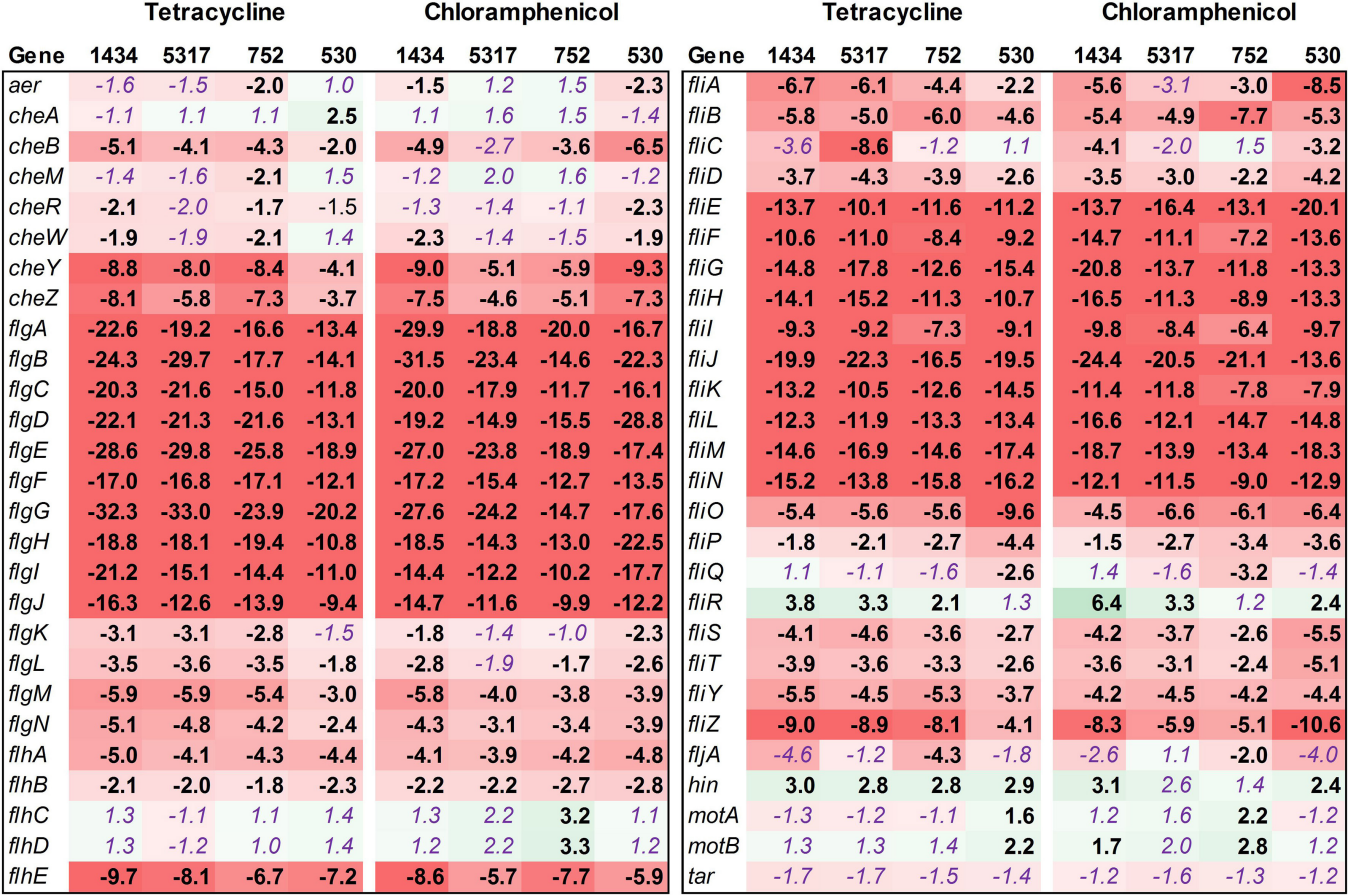
**RNA-seq analysis of genes associated with motility**. RNA-seq identified the fold-change in expression of genes associated with motility due to exposure to the antibiotics tetracycline (16 μg/ml) or chloramphenicol (64 μg/ml) when compared to the no-antibiotic control. Numbers in bold are significantly different (FDR-adjusted *P* < 0.05), while numbers in purple italics are not significant. Green denotes increased gene expression, and red indicates decreased gene expression; the intensity of the color highlights greater change.

Fifty-four genes associated with motility were assessed, and the majority of these were found to be down-regulated in isolates 1434, 5317, 752, and 530 when exposed to chloramphenicol and tetracycline (Figure 4, Table 3). This is highlighted by the *flgA-J* and *fliE-N* genes that encode proteins for the basal body, flagellar hook, and flagellar motor switch. The 10 *flg* and the 10 *fli* genes are all significantly down-regulated an average of 18.5-fold and 12.8-fold, respectively, in each of the four isolates in response to both antibiotics. The genes that encoded the master regulators of motility, *flhCD*, were not differentially regulated except in isolate 752 where their expression increased due to chloramphenicol. However, the *fliA* gene that encodes a σ^28^ factor and controls regulation of the late-flagellar genes (Chilcott and Hughes, 2000) was significantly down-regulated in most of the samples (the exception was isolate 5317 exposed to chloramphenicol that was down-regulated, but had a *P* = 0.054). Motility genes are typically up-regulated during early-log growth, and are then down-regulated during the mid- to late-growth phase as the invasion genes are up-regulated (Saini et al., 2010). In this study, we observed that tetracycline and chloramphenicol down-regulate the motility genes in *Salmonella* during early-log growth.

**Table 3:** **Mean and Standard Error of the Mean of all gene expression differences within each functional group after antibiotic exposure for isolates 1434, 5317, 752, and 530**.

	**1434**	**5317**	**752**	**530**
**TETRACYCLINE**				
Motility	−8.73 ± 1.15	−8.60 ± 1.16	−7.58 ± 0.97	−6.09 ± 0.89
SPI-1	10.21 ± 1.05	9.00 ± 1.25	2.47 ± 0.59	2.53 ± 0.40
SPI-2	13.82 ± 4.07	12.30 ± 2.96	7.39 ± 1.50	7.80 ± 1.55
SPI-3	24.17 ± 15.75	21.91 ± 12.92	18.93 ± 11.98	39.82 ± 27.93
Attachment	10.40 ± 2.07	10.44 ± 2.10	8.03 ± 1.37	5.43 ± 0.74
**CHLORAMPHENICOL**				
Motility	−8.66 ± 1.25	−6.80 ± 1.02	−5.92 ± 0.86	−8.09 ± 0.99
SPI-1	10.37 ± 1.38	8.35 ± 0.95	1.62 ± 0.62	3.89 ± 0.60
SPI-2	11.99 ± 2.29	10.36 ± 1.87	5.19 ± 1.08	4.84 ± 0.95
SPI-3	22.82 ± 14.18	23.80 ± 16.86	30.39 ± 24.22	23.61 ± 17.05
Attachment	11.33 ± 1.53	8.55 ± 1.06	5.97 ± 0.95	8.07 ± 1.33

#### SPI-1

**Figure 5:**
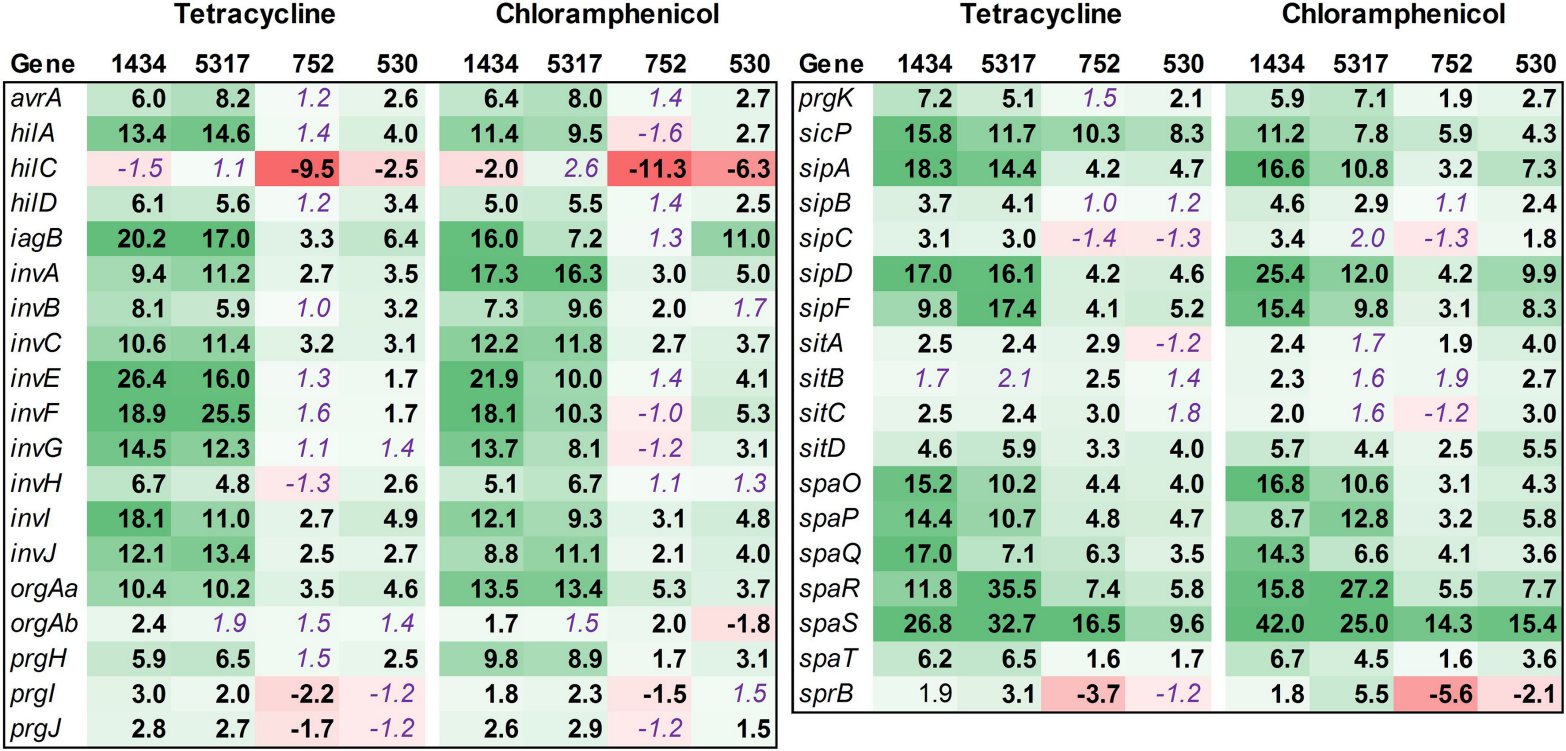
**RNA-seq analysis of SPI-1 genes**. RNA-seq identified the fold-change in expression of SPI-1 genes due to exposure to the antibiotics tetracycline (16 μg/ml) or chloramphenicol (64 μg/ml) when compared to the no-antibiotic control. Numbers in bold are significantly different (FDR-adjusted *P* < 0.05), while numbers in purple italics are not significant. Green denotes increased gene expression, and red indicates decreased gene expression; the intensity of the color highlights greater change.

Thirty-seven SPI-1 genes related to invasion were examined, which included those genes for SPI-1 regulation (*hil*), type-three secretion apparatus (*sip*, *spa*), and effector molecules (*inv*, *prg*) (Figure 5). While many of the SPI-1 genes are up-regulated in the four isolates, there is a striking difference between the two isolates associated with the induced invasion phenotype (1434 and 5317) and the other two isolates (752 and 530) (Table 3). This is denoted by the differences in the average fold-change between the two sets of isolates for all of the *inv* (12.4 ± 0.9 vs. 2.4 ± 0.3), *prg* (4.8 ± 0.7 vs. 0.7 ± 0.5), *sip* (10.5 ± 1.5 vs. 3.5 ± 0.7), and *spa* genes (16.0 ± 2.1 vs. 5.9 ± 0.8). Changes in *hilA* expression are consistent with the real-time data where 1434, 5317, and 530 had significant differences, but again the average fold-change for 1434 and 5317 was much higher than 530 (12.2 ± 1.1 vs. 3.3 ± 0.7, respectively). Thus, tetracycline and chloramphenicol can increase transcription of SPI-1 genes, but there may be a minimum threshold of induction that is necessary before the invasive phenotype is also induced.

#### SPI-2

**Figure 6:**
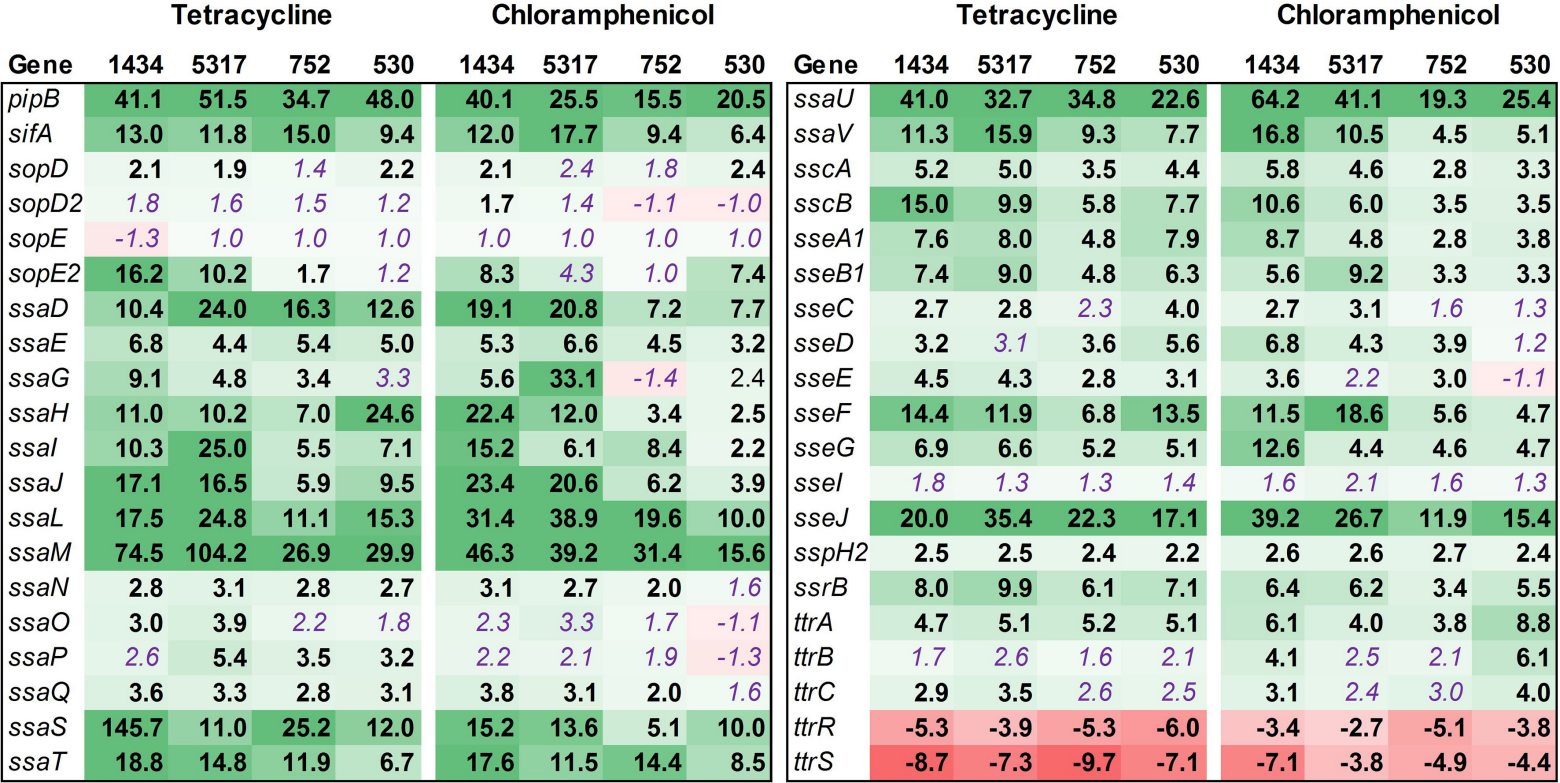
**RNA-seq analysis of SPI-2 genes**. RNA-seq identified the fold-change in expression of SPI-2 genes due to exposure to the antibiotics tetracycline (16 μg/ml) or chloramphenicol (64 μg/ml) when compared to the no-antibiotic control. Numbers in bold are significantly different (FDR-adjusted *P* < 0.05), while numbers in purple italics are not significant. Green denotes increased gene expression, and red indicates decreased gene expression; the intensity of the color highlights greater change.

SPI-2 encodes a TTSS that is required for intracellular replication and survival, and of the 40 genes examined in this functional group, the majority were up-regulated (Figure 6). The bias in up-regulation that appeared in SPI-1 for isolates 1434 and 5317 was also present in SPI-2 (Table 3). Several genes encoding effector molecules, such as *sifA*, *pipB*, and *sseJ*, were up-regulated between 6.4 and 51.5-fold for the four isolates exposed to either antibiotic. Only two genes were significantly down-regulated, *ttrR* and *ttrS*, and these encode a two-component regulatory system for reducing tetrathionate.

#### SPI-3

**Figure 7:**
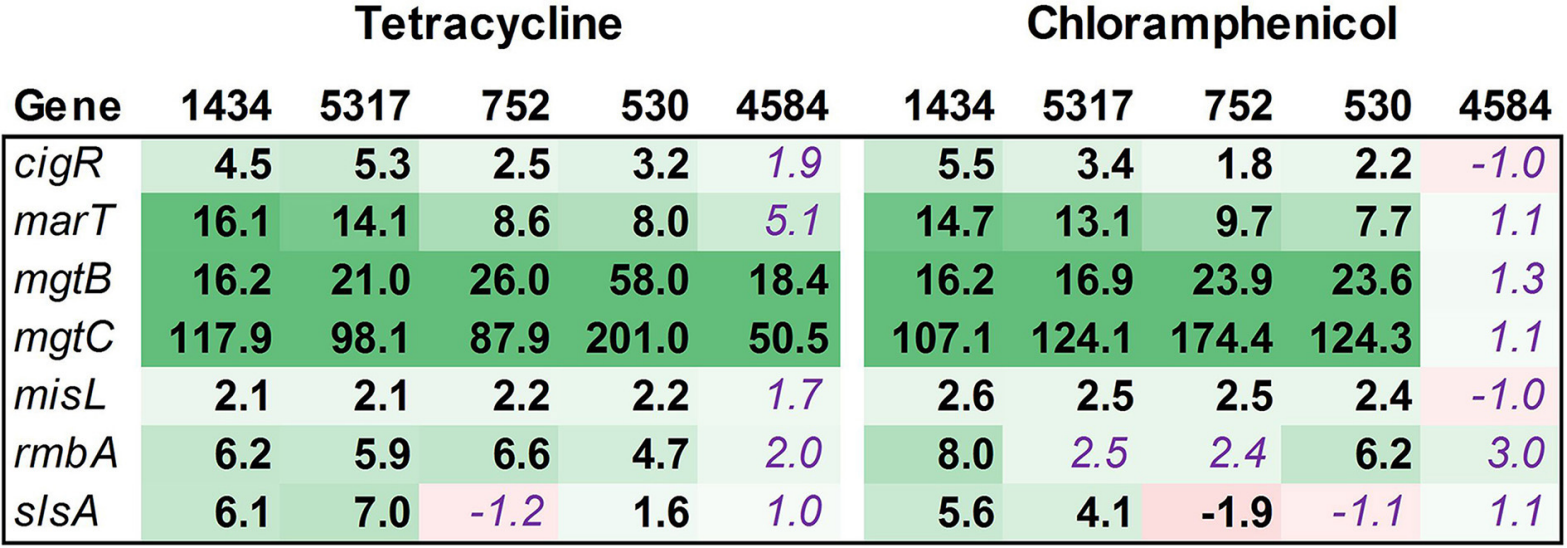
**RNA-seq analysis of SPI-3 genes**. RNA-seq identified the fold-change in expression of SPI-3 genes due to exposure to the antibiotics tetracycline (16 μg/ml) or chloramphenicol (64 μg/ml) when compared to the no-antibiotic control. Numbers in bold are significantly different (FDR-adjusted *P* < 0.05), while numbers in purple italics are not significant. Green denotes increased gene expression, and red indicates decreased gene expression; the intensity of the color highlights greater change.

The SPI-3 region has few genes, but these play a role in virulence and intracellular survival in monocytes. Most of the SPI-3 genes were up-regulated (Figure 7), including *mgtBC*. MgtB is used by *Salmonella* to transport magnesium into the bacterium in magnesium-deficient environments such as an intracellular environment. The tetracycline and chloramphenicol efflux pumps are major facilitator superfamily antiporters that export an antibiotic:proton complex outside of the cell (Chopra and Roberts, 2001; Braibant et al., 2005). It is known that tetracycline molecules bind to magnesium, and the export of this complex would therefore decrease the internal concentration of the ion. It would be beneficial for *Salmonella* to up-regulate this system in order to continue to export the antibiotic. The *mgtB* and *mgtC* genes are up-regulated in isolate 4584 exposed to tetracycline but not chloramphenicol, and these are the only virulence-associated genes examined in this study to be differentially regulated in this isolate.

#### Attachment

**Figure 8:**
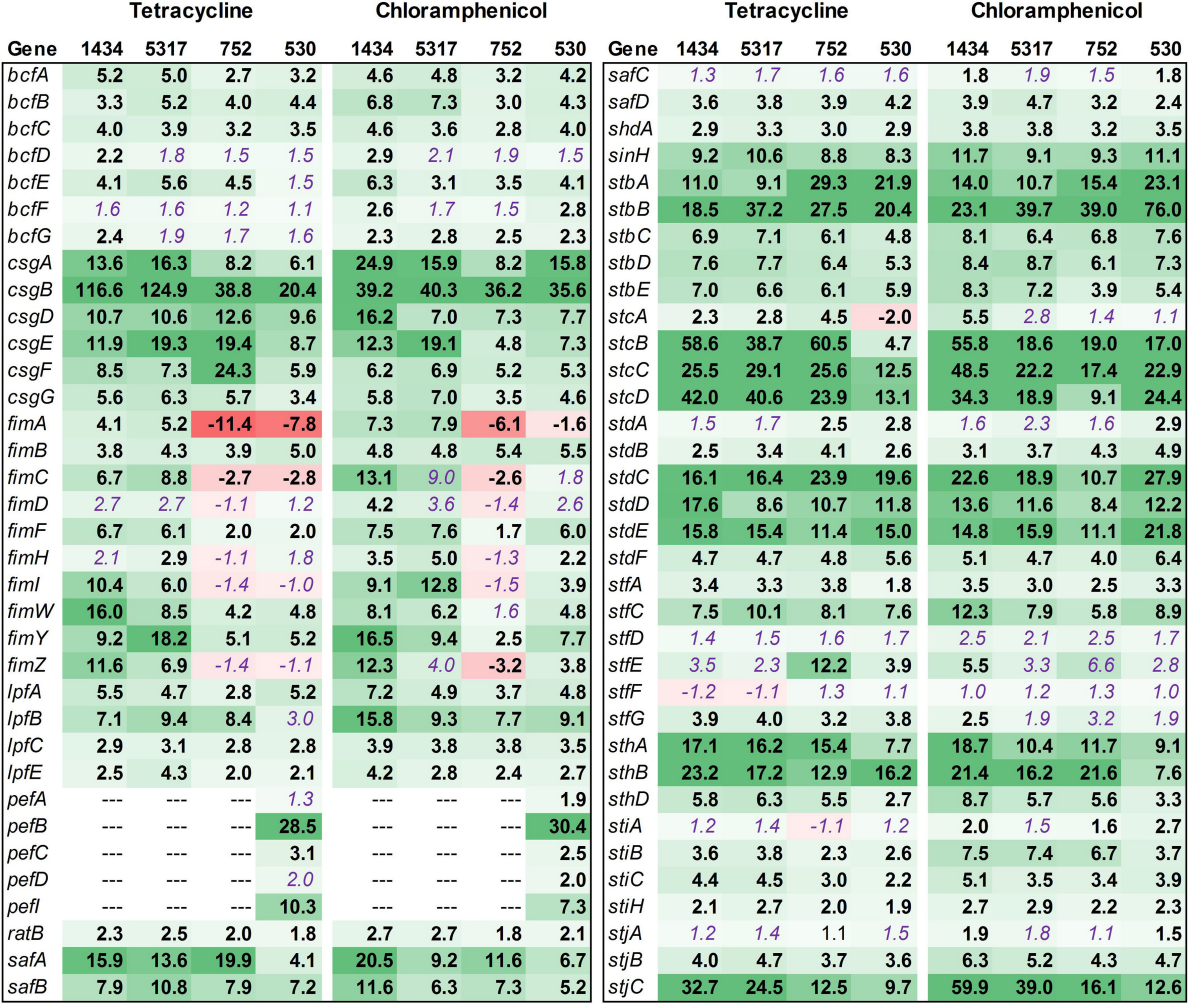
**RNA-seq analysis of genes associated with attachment**. RNA-seq identified the fold-change in expression of genes associated with attachment due to exposure to the antibiotics tetracycline (16 μg/ml) or chloramphenicol (64 μg/ml) when compared to the no-antibiotic control. Numbers in bold are significantly different (FDR-adjusted *P* < 0.05), while numbers in purple italics are not significant. Green denotes increased gene expression, and red indicates decreased gene expression; the intensity of the color highlights greater change.

*Salmonella* Typhimurium isolates encode up to 13 fimbrial operons (*bcf*, *csg*, *fim*, *lpf*, *pef*, *saf*, *stb*, *stc*, *std*, *stf*, *sth*, *sti*, and *stj*). These genes, as well as other genes related to attachment, were evaluated (Figure 8). Overall, most of the fimbrial operons were up-regulated in each isolate due to antibiotic exposure. However, there were a few notable differences among the isolates. Most of the *pef* genes were up-regulated in 530 (DT104) whereas the three DT193 isolates (1434, 5317, and 752) lacked this operon. Isolates 1434 and 5317 had a greater level of *fim* gene expression compared to isolates 752 and 530. The *fim* genes encode the Type I fimbriae, which are important for *Salmonella* entry into epithelial cells (Ernst et al., 1990; Baumler et al., 1996; Wilson et al., 2000). The increased *fim* expression in isolates 1434 and 5317 may also play a role in the induced invasion phenotype observed for these two isolates.

Previously, only three fimbrial operons (*csg*, *fim*, and *pef*) had been shown to be expressed in culture, and each requires specific growth conditions (Humphries et al., 2003). Typically, expression of *csg* is dependent on growth for over 24 h on solid medium, while *fim* expression occurs in the stationary phase for *Salmonella* grown in liquid medium. The *pef* genes are encoded on a plasmid and require liquid medium and a low pH to be expressed in culture. The expression of the remaining fimbrial genes has only been observed *in vivo* (Nicholson and Baumler, 2001; Humphries et al., 2003). However, in the current study, tetracycline and chloramphenicol induced the expression of genes in up to 11 different fimbrial operons in isolates 1434, 5317, 752, and 530. Also, the fimbrial genes in the *bcf*, *lpf*, *stb*, *stc*, *std*, and *sth* operons have been shown to be associated with persistence in mice (Weening et al., 2005); as all six of these operons can be induced by the two antibiotics, this could be one factor that contributes to the increased morbidity observed in MDR *Salmonella* patients that have been treated with antibiotics (Molbak, 2005).

### Electron microscopy demonstrates decreased flagella and increased agglutination due to antibiotic exposure

**Figure 9:**
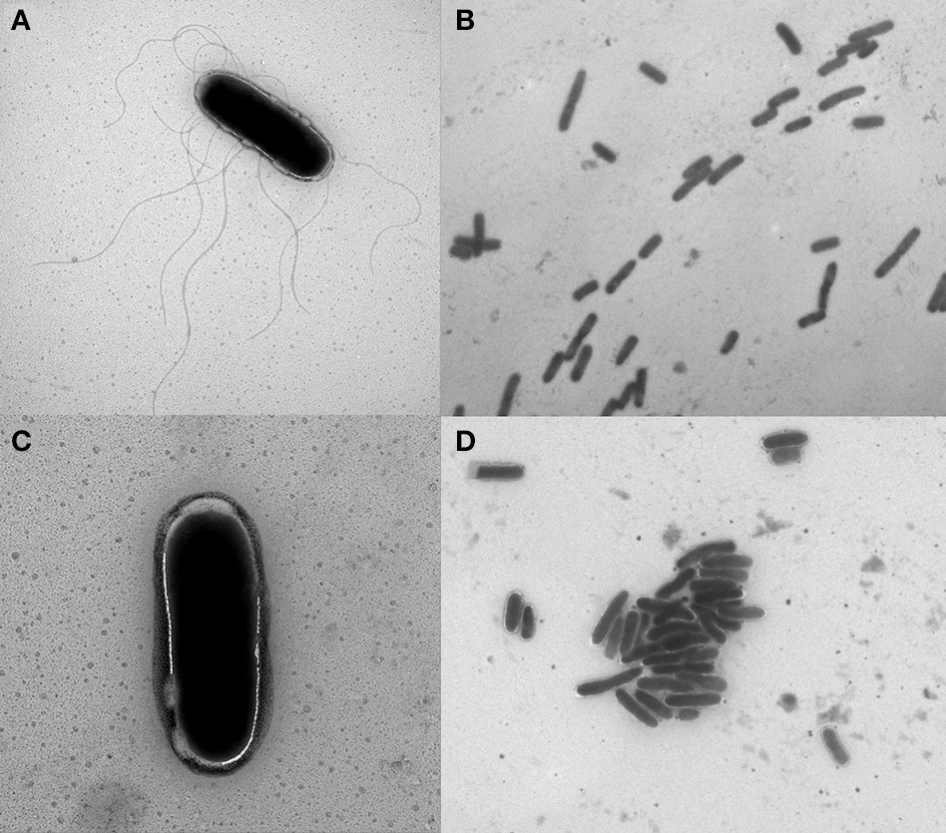
**Electron micrographs of *Salmonella* with and without chloramphenicol exposure**. *S*. Typhimurium isolate 1434 grown to early-log phase (OD_600_ = 0.15) and exposed to **(A,B)** 0 μg/ml or **(C,D)** 64 μg/ml chloramphenicol for 30 min. Isolates exposed to chloramphenicol had fewer flagella and increased bacterial agglutination compared to the control (no antibiotic) condition. These figures are representative of other regions of the samples that were also visualized.

Electron microscopy was used to visualize isolate 1434 in early-log phase treated with 64 μg/ml chloramphenicol compared to a no antibiotic control (Figure 9). *Salmonella* that were not exposed to chloramphenicol (control) had many flagella (Figure 9A) and were dispersed (Figure 9B). The chloramphenicol-treated *Salmonella* had few-to-no flagella (Figure 9C) and had increased bacterial agglutination compared to the untreated sample (Figure 9D). These figures are representative of other regions of the samples that were also visualized. Isolate 1434 exposed to 16 μg/ml tetracycline had the same result (data not shown). The decreased flagella and increased agglutination are consistent with the RNA-seq data that demonstrates decreased motility and increased attachment gene expression.

### Concluding remarks

Tetracyclines are approved for growth promotion and therapeutic use in swine, cattle, and poultry in the U.S.; it was reported that over 4.6 million kg of tetracyclines were sold for use in livestock in a single year (USGAO, 2011). While tetracycline usage may directly select for resistant *Salmonella* encoding *tet* genes, it may also indirectly select for other resistance genes (such as *floR*) and virulence factors that are encoded on the same plasmid. For example, Hradecka et al. reported the *Salmonella* virulence plasmid can recombine with an antibiotic resistance plasmid, ensuring that the plasmid and its content can be selected based on either virulence or antibiotics (Hradecka et al., 2008). Because of the widespread use of antibiotics in humans and livestock, it is important to understand the effects these antibiotics have on MDR *Salmonella*.

Tetracycline and chloramphenicol exposure of MDR Salmonella in our transcriptomic data indicate that the regulation of the invasive phenotype in *Salmonella*, which normally takes over 8 h to induce, can be accelerated to occur in 30 min. While gene expression data cannot assess protein levels or post-transcriptional interactions of each individual gene that have an impact on the invasion phenotype, the data suggests there is a coordinated regulation of the regulons involved in attachment, motility, and invasion due to chloramphenicol and tetracycline. Furthermore, the attachment regulon appears to extend beyond the *fim* operon to include other fimbrial operons that would not normally be expressed in culture. Chloramphenicol and tetracycline may be acting on a complex stimulon with multiple interacting regulons in a system that is currently uncharacterized in culture.

The induced invasion phenotype observed may be directly activated by the antibiotic response pathway that is stimulated to express the efflux pump after antibiotic exposure. It may also be an indirect response due to the changes in the cellular environment due to the presence of the antibiotic and potential changes in H^+^ and Mg^2+^ concentrations (Yamaguchi et al., 1990). For instance, the response may be due to the “tetracycline transport cycle” in resistant cells where extracellular tetracycline is protonated and diffuses into the bacterial cell in a neutral form. Once inside the cell, the tetracycline is predicted to dissociate from the proton due the higher intracellular pH. The intracellular concentration of Mg may facilitate the formation of a tetracycline-Mg chelate complex that can then be exported by the TetA antiporter with the simultaneous import of a proton. In this model, the export of tetracycline is electrically neutral (Mg^+^ exported while H^+^ imported) but there is a net gain of one proton from the initial diffusion of tetracycline into the bacterial cell. Therefore, the internal pH of the bacteria may shift due to the accumulation of protons with the decreased pH conditions triggering a secondary response that activates the invasion genes. Interestingly, *mgtBC* present in SPI-3 is up-regulated by both tetracycline and chloramphenicol exposure. The MgtC protein inhibits the bacterium's own F_1_F_0_ ATP synthase that couples proton translocation across the bacterial membrane with ATP synthesis/hydrolysis (Lee et al., 2013). The intracellular pH is lower and ATP levels higher for an *mgtC* mutant compared to wild-type *S*. Typhimurium suggesting that intracellular pH or ATP/ADP levels could influence the gene expression seen in our investigation. Because the induced invasion phenotype does not occur in all the MDR isolates tested, there is likely a genetic component that is involved. Additional investigations using genomics and proteomics will provide greater insights into the effect antibiotics have on these MDR *Salmonella* isolates.

### Conflict of interest statement

The authors declare that the research was conducted in the absence of any commercial or financial relationships that could be construed as a potential conflict of interest.

## Abbreviations

MDR: multidrug-resistant
FDR: false discovery rate
SPI: *Salmonella* pathogenicity island
TTSS: type three secretion system.
